# Near-Infrared Activated Cyanine Dyes As Agents for Photothermal Therapy and Diagnosis of Tumors

**DOI:** 10.32607/actanaturae.11028

**Published:** 2020

**Authors:** E. I. Shramova, A. B. Kotlyar, E. N. Lebedenko, S. M. Deyev, G. M. Proshkina

**Affiliations:** Shemyakin-Ovchinnikov Institute of Bioorganic Chemistry, Russian Academy of Sciences, Moscow, 117997 Russia; Tel Aviv University, Ramat Aviv, Tel Aviv, 69978 Israel; National Research Tomsk Polytechnic University, Tomsk, 634050 Russia

**Keywords:** cyanines, near infrared, photothermal therapy

## Abstract

Today, it has become apparent that innovative treatment methods, including those involving simultaneous diagnosis and therapy, are particularly in demand in modern cancer medicine. The development of nanomedicine offers new ways of increasing the therapeutic index and minimizing side effects. The development of photoactivatable dyes that are effectively absorbed in the first transparency window of biological tissues (700–900 nm) and are capable of fluorescence and heat generation has led to the emergence of phototheranostics, an approach that combines the bioimaging of deep tumors and metastases and their photothermal treatment. The creation of near-infrared (NIR) light-activated agents for sensitive fluorescence bioimaging and phototherapy is a priority in phototheranostics, because the excitation of drugs and/or diagnostic substances in the near-infrared region exhibits advantages such as deep penetration into tissues and a weak baseline level of autofluorescence. In this review, we focus on NIR-excited dyes and discuss prospects for their application in photothermal therapy and the diagnosis of cancer. Particular attention is focused on the consideration of new multifunctional nanoplatforms for phototheranostics which allow one to achieve a synergistic effect in combinatorial photothermal, photodynamic, and/or chemotherapy, with simultaneous fluorescence, acoustic, and/or magnetic resonance imaging.

## INTRODUCTION

The phototherapy of tumors using organic compounds dates back to 1972, when experiments by I. Diamond and colleagues on rats showed the promise of hematoporphyrin as a powerful phototherapeutic agent for selective destruction of glioma cells [1]. Since then, a large number of organic compounds based on porphyrin, cyanine, and polymer dyes have been developed for phototherapy, some of which are used in medical practice today [2, 3].

This review is devoted to the use of organic infrared (IR) dyes as agents for the photothermal therapy and diagnosis of tumors. The theoretical aspects of phototherapy and the physicochemical properties of the agents used in phototherapy are described in detail in reviews [4, 5, 6, 7].

Phototherapy is based on a selective destruction of tumor cells under the influence of light. Dyes absorb light and convert its energy into heat, thereby causing cell damage and death. Phototherapy with dyes includes photodynamic therapy (PDT) and photothermal therapy (PTT). In the case of PDT [7], light induces chemical reactions, the products of which have a negative effect on the vital activity of cells. In the case of PTT [8], the dye directly transforms light energy into heat, causing thermal damage to cells.

**Fig. 1:**
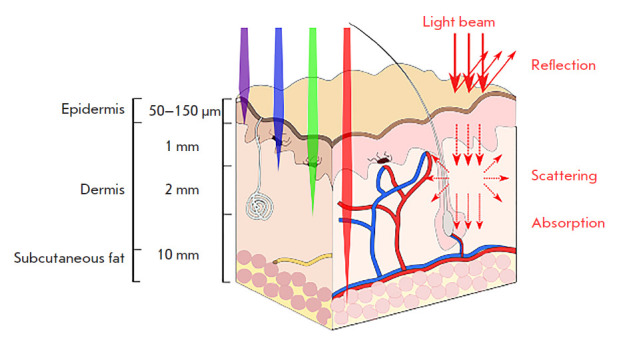
Depth of light penetration of human tissues

**Fig. 2:**
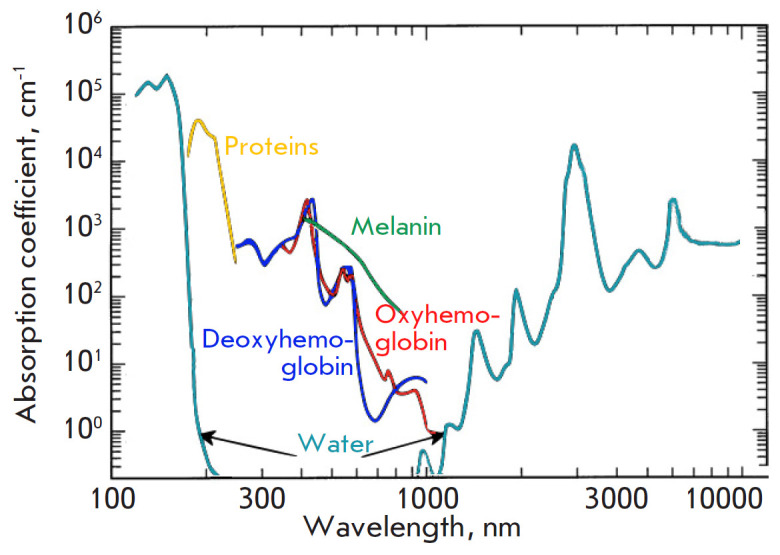
Visible and infrared absorption spectra of biological tissues (adapted from [9])

Due to the intense absorption of visible and ultraviolet light quanta by biological tissues (*Fig. 1*), phototherapy with light in an indicated range is used in clinical practice only to treat superficial tumors exposed to external light sources. It is known that proteins, nucleic acids, vitamins, and most cofactors efficiently absorb in the ultraviolet region of the spectrum; oxyhemoglobin, deoxyhemoglobin, and melanin intensively absorb in the visible region of the spectrum (400–650 nm). Therefore, the preferred excitation wavelengths in medicine (transparency window) are near-IR light in a range of 700–900 nm [4]. Light in a range of 900 to 1,100 nm cannot be used due to the strong absorption of water (*Fig. 2*).

Phototherapy has several obvious advantages, including non-invasiveness, ability to affect deep body tissues, small area and accuracy of irradiation, and regulation of the degree of tumor exposure via changing of the irradiation dose. In addition to these advantages, when using near-infrared light in phototherapy, the excitation light penetrates deeply into biological tissues and causes less background fluorescence; also, infrared dyes are characterized by extremely rare activation by visible light.

In recent years, phototherapy has significantly advanced thanks to the use of lasers as light sources; nano-objects for the delivery of sensitizers [10-13]; targeted dyes [14, 15]; increased dye circulation time in the blood [16]; and sustained release of dyes [17]. Also, conjugation of dyes with immunoadjuvants is promising in photoimmunotherapy because it leads to the triggering of a systemic immune response [18].

Hypoxia is well known as a distinctive feature of solid tumors [19]. That is why the phototherapy of such tumors should use substances that act not photodynamically, but photothermally.

**Fig. 3:**
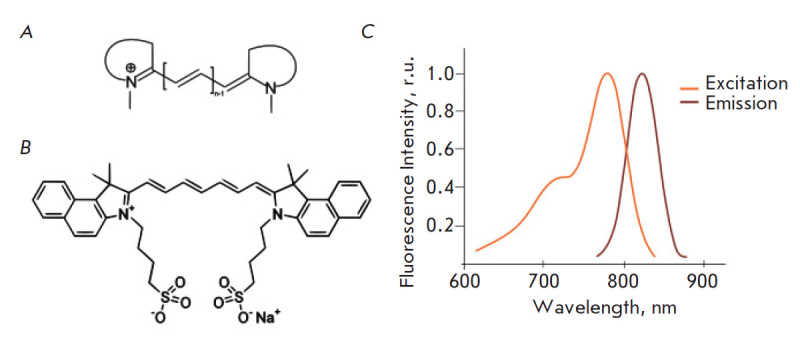
General structure of cyanine dyes (*A*); structure of indocyanine green (ICG) (*B*); excitation and emission spectra of ICG (*C*)

Today, PTT is a clinically approved technique that is used to treat patients with solid tumors of the liver, kidneys, lungs, adrenal glands, prostate, and bones [20]. An increase in the tumor temperature of up to 42°C renders cancer cells more susceptible to traditional treatment techniques (radiotherapy and chemotherapy), because an increase in temperature enhances the permeability of biological membranes and accelerates endocytosis and blood circulation [21]. An increase in tissue temperature to 45°C or above leads to necrosis of tumor cells [22].

In recent years, nanotechnology has been actively used to develop photothermal sensitizing agents, such as gold nanoparticles [23], gold nanorods [10], upconverting nanoparticles [14, 24-28], carbon nanotubes, graphene and its derivatives, and many others [8].

In biomedical imaging and phototherapy, organic dyes, thanks to their versatile photophysical properties and simplicity of large-scale synthesis, hold a special place among photoactivatable agents. Organic dyes can be conjugated to various specific biomolecules, which expands the range of their applications for therapy. The disadvantage of most dyes is their instability and rapid elimination from the bloodstream.

Photothermal agents should exhibit several basic properties, such as: 1) strong absorption in the near infrared region (extinction coefficients > 1 × 10^5^ M^-1^cm^-1^); 2) biocompatibility and biodegradability; and 3) real-time imaging to control therapy [29]. Cyanine-based dyes, which are widely used in the phototherapy of tumors, fully possess these properties. Cyanines are synthetic organic dyes (*Fig. 3A*) that are excited by infrared light (780–820 nm) and excellent for fluorescence imaging and phototherapy.

## INDOCYANINE GREEN AND NANOSYSTEMS FOR ITS DELIVERY

Indocyanine green (ICG) (*Fig. 3 B,C*) is a carbocyanine dye widely used in medical diagnostics [30]. Thanks to its spectral characteristics, this dye can be used as a contrast agent for optical imaging in angiography [31, 32], the biopsy of sentinel lymph nodes in breast cancer [33], assessment of blood plasma volume after cardiovascular surgery [34, 35], and evaluation of the functional reserves of the liver in hepatology [36]. Also, ICG is one of the least toxic contrast agents approved for use in medical practice [37]. The only known adverse reaction to ICG is anaphylactic shock in rare cases [38]. Under the action of an IR laser (λ = 808 nm; radiation flux density, 155 W/cm2), ICG converts most of the excitation energy into heat and, after 30 s of irradiation, causes local heating of the tissue to 75°C [39]. In this case, part of the energy is spent on the production of singlet oxygen, so ICG can be used for combined photothermal (PTT) and photodynamic therapy (PDT) [40].

After intratumoral injection, ICG was shown to accumulate well in tumor tissues and sentinel lymph nodes [41]. As shown in *in vitro *experiments, irradiated ICG induces the death of squamous cell carcinoma [42], colon cancer [43], and human pancreatic cancer [44] cells.

ICG has a low quantum yield of fluorescence [45, 46] and is susceptible to photobleaching, which limits its use in long-term bioimaging *in vivo *[39, 47, 48]. Many researchers have noted that ICG mo are oxidized and dimerized in an aqueous medium, which leads to decreased absorption of exciting light, reduced fluorescence, and a maximum absorption wavelength shift [30, 49, 50-55]. In addition, upon systemic administration, ICG cannot specifically accumulate in tumors because it quickly binds to blood plasma albumin and is rapidly excreted from the body (2–4 min) [49, 52, 56].

Various nanocarrier systems have been developed to increase the circulation time of ICG in the body. For example, to date, ICG-containing nanoparticles have been developed based on polymeric complexes [57, 58], peptides [59], proteins [60, 61, 62], micelles [63, 64], magnetic [65] and polylactide glycolide (PLGA) [66] particles. Encapsulation of the dye in PLGA particles improved the stability of ICG in water and increased its thermal stability [66]. Eight-hour incubation of PLGA particles under physiological conditions resulted in 78% dye leakage. To overcome this problem, silica polymer composite microcapsules were developed. This resulted in a 17% reduction in ICG leakage [39], but it required increasing the particle size to 1 μm. In addition, polymeric shells were found not to protect encapsulated dye molecules from dimerization or photoisomerization, as evidenced by an absorption peak wavelength shift to longer wavelengths [39, 67, 68] and a significant decrease in the fluorescence peak intensity [66]. The properties of encapsulated ICG molecules were improved by using organically modified silicates as carriers [69]; however, even in this case, the sizes of the produced particles were not small enough and amounted to about 100 nm, which corresponds to the upper size limit of the carriers used in *in vivo *experiments [70].

Several studies have proposed biodegradable calcium phosphate nanoparticles as ICG carriers for therapy and bioimaging [71, 72, 73]. The mean particle size in suspension is about 16 nm, and functionalization of the outer particle surface with carboxylate or polyethylene glycol (PEG) preserves the stability of the particles in physiological solutions for a long time and simultaneously preserves the high quantum yield and photostability of the dye. Upon intravenous administration, ICG-loaded particles coated with PEG were shown to accumulate, due to increased capillary permeability and impaired lymphatic drainage in tumor tissue, in xenografted tumors of model animals; in this case, the dye was detected *in vivo *within four days after its administration. In this case, the surface of the loaded nanoparticles can be functionalized with targeted antibodies to enhance the directed accumulation of particles in the tumor, which was demonstrated in breast tumors via targeting of the transferrin receptor CD71 [72]; pancreatic cancer cells via targeting of the gastrin receptor [72]; and leukemia cells via targeting of the receptor tyrosine kinase CD117 and type I transmembrane glycoprotein CD96 [73].

The ICG–polyethyleneimine (PEI) complexes incorporated into silicon dioxide nanoparticles [74] had improved photophysical properties compared to those of the dye. The interaction with PEI prevented ICG aggregation and quenching of dye fluorescence, and it stopped dye leakage from the particles. The use of an ICG–PEI complex in combination with silicon nanoparticles enabled detection of IR signals at a depth of up to 2 cm from the body surface during bioimaging. The interaction between ICG and proteins changes the dye fluorescence parameters, a property used to create targeted IR probes. After binding to receptors and internalization, the dye dissociated from antibodies, which led to a restoration of the initial parameters of dye fluorescence. Targeted probes have been developed based on ICG complexes with daclizumab, trastuzumab, or panitumumab, which interact with interleukin-2 (CD25) receptors and human epidermal growth factor II and I (HER2 and HER1) receptors, respectively [75].

**Fig. 4:**
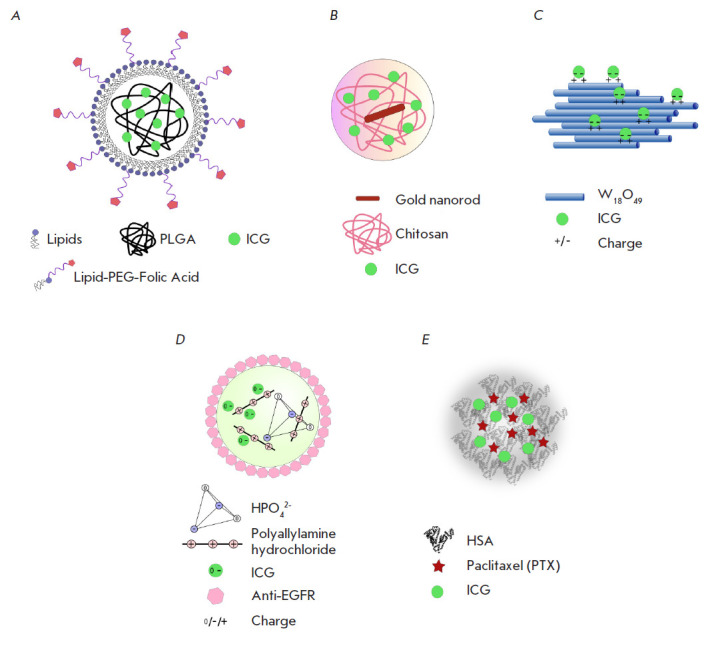
Nanosystems for the delivery of ICG to tumor cells. *A *– folic acid-functionalized multilayer lipid nanoparticles loaded with ICG [76]; *B *– chitosan nanospheres with encapsulated gold nanorods and ICG [77]; *C *– wolfram oxide nanorods with surface-bound ICG [78]; *D *– polyallylamine hydrochloride–phosphoric acid salt nanospheres loaded with ICG and functionalized with anti-human epidermal growth factor receptor (EGFR) antibodies [57]; *E *– self-organized nanoparticles consisting of human serum albumin (HSA), paclitaxel (PTX), and ICG [79]

Targeted delivery of ICG into cells by lipid nanoparticles functionalized with folic acid molecules (*Fig. 4A*) is an alternative method for targeted delivery of a dye into cells [76]. These biocompatible particles were found to have good monodispersity, retain photostability, and to be characterized by a longer circulation time in the bloodstream compared to that of free ICG. *In vivo *experiments confirmed the targeted uptake of the described particles by the tumor, which makes lipid nanoparticles ideal agents for intravital bioimaging and early cancer diagnosis.

For bimodal phototherapy combining both PTT and PDT, a nanoplatform based on hybrid chitosan nanospheres with encapsulated gold nanorods and ICG was proposed (*Fig. 4B*) [77]. The hybrid nanospheres had a diameter of 180 nm and absorbed in a range of 650 to 900 nm. ICG inside the nanospheres was protected from rapid hydrolysis in biological fluids, which increased the dye’s lifetime and its effect on the cells. Bimodal phototherapy demonstrated a high synergistic effect and improved the therapeutic efficacy of either ICG or gold nanorods alone. For example, after the irradiation of nanosphere-pretreated model animals with an IR laser, the tumor volume increased only 16-fold in mice of the experimental group and 58-fold in mice of the control group.

Tungsten oxide (W_18_O_49_) nanorods and ICG can also be used for bimodal phototherapy [78]. In such a design, tungsten oxide nanorods simultaneously act as an effective photothermal agent for PTT and as a nanocarrier that electrostatically binds ICG molecules on its surface (*Fig. 4C*). As in the case of gold nanorods, bimodal therapy triggered by irradiation of tungsten oxide nanorods was accompanied by increased lethality of HeLa cells compared to monomodal therapy (PTT or PDT alone). Experiments on animals have shown that tungsten nanorods with bound dye molecules effectively destroy solid tumors when exposed to light (808 nm), thus demonstrating the high potential of these nanocomposites in tumor therapy.

The use of ICG spherical composite capsules consisting of polyallylamine hydrochloride molecules and orthophosphoric acid salts (*Fig. 4D*) for PTT was reported in [57]. The capsule surface was functionalized with anti-human epidermal growth factor receptor (EGFR) antibodies targeting EGFR-positive cancer cells. In *in vitro *experiments, the irradiation of cells with an IR laser (808 nm) with an irradiation intensity of 6 W/cm2 caused almost 100% death of cells treated with anti-EGFR nanocapsules loaded with ICG, while the death rate of cells treated with a free dye amounted to only 15%.

A nanotheranostic platform consisting of three clinically approved agents, human serum albumin (HSA), paclitaxel (PTX), and ICG, was developed for PTT and bioimaging (*Fig. 4E*) [79]. Mixing of HSA, PTX, and ICG molecules was shown to lead to the formation of stable 80 nm nanoparticles. In this system, HSA plays the role of a biocompatible carrier, PTX is an effective antitumor drug, and ICG acts both as a probe for fluorescence imaging and as a photothermal agent. These three-component nanoparticles (HSA–ICG–PTX) were shown to possess higher stability and a more extended lifetime in the bloodstream than the HSA–ICG complex. Moderate photothermal heating caused by irradiation of ICG with an IR laser increases the intracellular uptake of HSA–ICG–PTX, which enhances the cytotoxicity of the complex. *In vivo *experiments using intravital bioimaging have demonstrated that nanocomplexes efficiently accumulate in the primary tumor and lung metastases. In the case of subcutaneous tumors and metastases, therapy with three-component nanoparticles produces an excellent synergistic effect based on chemical and photothermal effects. The described theranostic nanoplatform, which consists of clinically approved agents, is very promising for both non-invasive detection of a disease focus and treatment of oncological diseases.

Targeted liposome particles loaded with a dye and superficially functionalized with folic acid [80] were successfully used to suppress MCF-7 human breast adenocarcinoma cells overexpressing folate receptors on their surface. These liposomal particles were shown to be effective in PTT *in vitro *and *in vivo*.

## INDOCYANINE GREEN ANALOGS WITH IMPROVED PROPERTIES

**Fig. 5:**
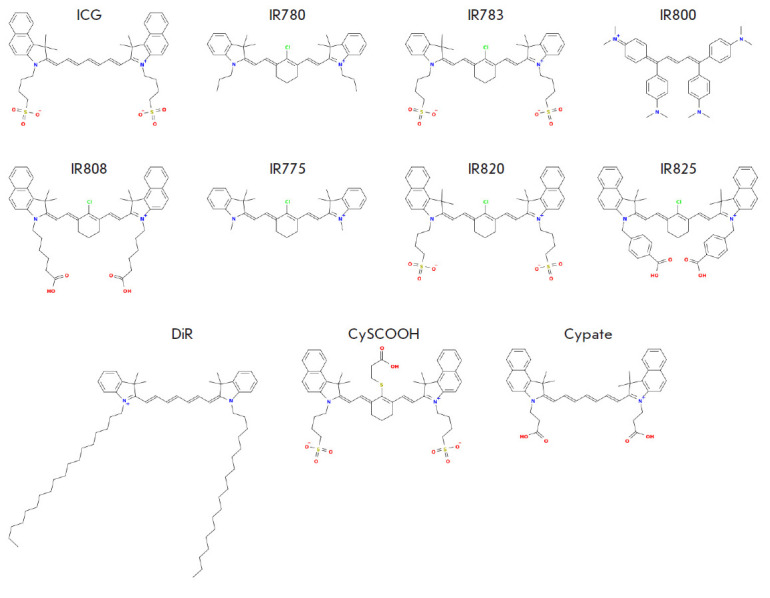
Structures of cyanine dyes (ICG analogs)

Along with ICG, recent studies have used a number of dye analogs that are characterized by improved photo-optical properties and increased stability in biological media [81, 82] (*Table*, *Fig. 5*).

IR780, IR783, IR800, and IR808 dyes have been successfully used for bioimaging [86, 93-96]. IR780, IR783, and IR808 water-soluble dyes were found to preferentially accumulate in tumor cells *in vitro *and *in vivo*. However, like ICG, they are rapidly cleared from the bloodstream and are characterized by short retention in the tumor, which limits the time window for phototherapy [97].

IRDye800CW (IR800) is a water-soluble analog of ICG. It is approved for clinical use and used for biomedical imaging and fluorescence surgery, a technique involving a fluorescent contrast agent to improve intraoperative tumor imaging [96, 98]. Conjugates of IR800 with various antibodies targeting growth factors and proteoglycans have been successfully used in preclinical and clinical trials for phototheranostics of brain tumors [96, 99, 100, 101], breast cancer [102], and head and neck cancer [103, 104, 105].

The use of highly efficient hydrophobic analogs of ICG required the development of systems for delivery of the dyes to the disease focus, based on various nanocarriers [106, 107]. For example, in 2017 [108], a phototheranostic nanoplatform based on a hydrophobic analog of ICG, IR775, was developed for bimodal therapy (PDT and PTT) in combination with real-time bioimaging. Water-insoluble IR775 was loaded into 40-nm biocompatible PEG–polycaprolactone polymeric nanoparticles for delivery to tumors. Nanoparticle-encapsulated IR775 causes heating of a test liquid up to 55°C and triggers production of reactive oxygen species upon irradiation. *In vivo *experiments have shown that, after systemic administration, nanoparticle-encapsulated IR775 efficiently accumulates in cancerous tumors, produces a clear fluorescent signal upon IR irradiation, and leads to complete destruction of a tumor resistant to traditional chemotherapy after only a single session of combinatorial phototherapy.

For multimodal PTT with simultaneous fluorescence and photoacoustic imaging, a theranostic nanoplatform based on ferritin nanoparticles loaded with IR820, called “chameleon,” was developed [62]. The absorption spectrum of free IR820 contains a minor peak at 550 nm. Excitation of both the free and particle-encapsulated versions of the dye with a light source at 550 nm produced an emission with a maximum at 604 nm. Excitation of the dye at a main absorption peak wavelength (770 nm) resulted in an emission with a maximum at 834 nm. This property of IR820 enabled excitation of nanoparticles at 550 nm for fluorescence imaging and excitation with an IR laser at 808 nm for photoacoustic imaging and highly efficient PTT. Intravenous injection of nanoparticles to model animals, followed by low intensity (0.5 W/cm2) IR irradiation, resulted in complete disappearance of tumors without significant toxicity or relapses.

**Fig. 6:**
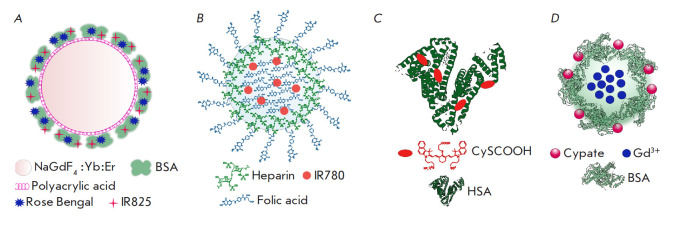
Multifunctional platforms based on ICG dye analogs for phototheranostics. *A *– upconverting nanoparticles with bovine serum albumin (BSA) incorporating Rose Bengal and IR825 [109]; *B *– heparin and folic acid-based nanoparticles loaded with IR780 [58]; *C *– conjugates of human serum albumin (HSA) and CySCOOH [91]; *D *– gadolinium nanoparticles coated with a BSA-Cypate conjugate [113]

Combination therapy also uses upconverting nanoparticles (UCNPs) (*Fig. 6A*). To increase solubility and stability in physiological fluids, UCNPs were coated with bovine serum albumin (BSA). In this case, two

**Table:** Basic photophysical properties of ICG and its IR analogs

	IR dye	Absorption λ_max_, nm	Emission λ_max_, nm	Extinction coefficient, ε (× 10^5^ M^−1^cm^−1^)	Quantum yield of singlet oxygen, %*	Quantum yield of fluorescence, %	Reference
1	ICG	785	822	2.04	0.8	7.8M	[82]
2	IR780	780	798–823**	2.65	12.7	0.07–0.17%**	[83, 84]
3	IR783	783	804	1.17	3	4	[82]
4	IR800	774	794	2.40	N/D	9	[85]
5	IR808	783M	816	3.00	N/D	5.9	[86, 87]
6	IR775	775M	792	2.37	N/D	7	[88]
7	IR820	820	850	2.02	2	4.4	[82]
8	IR825	825M	–	1.14	N/D	< 0.1M	[89]
9	DiR	747M	774M	2.70	N/D	28	[90]
10	CySCOOH	820	840	N/D	N/D	N/D	[91]
11	Cypate	785	822	2.16	2	6.5	[92]

^*^Relative to Rose Bengali [82];

^**^depending on the solvent;

N/D – no data;

M – in methanol.

On the basis of micelles loaded with the IR780 dye and radioactive isotope rhenium-188 (188Re), a multifunctional platform was developed for PTT, fluorescence imaging, and single-photon emission tomography [110]. This platform enables real-time monitoring of the accumulation and distribution of micelles in the tumor, as well as the release kinetics of the drugs loaded into the micelles. In *in vivo *PTT experiments on model animals with xenograft tumors (rectal cancer), inhibition of tumor growth was achieved in 82.6% of the animals of the experimental group. A histopathological analysis revealed irreversible necrotic tissue damage, decreased proliferative activity, enhanced cell apoptosis, and increased expression of heat shock proteins in tumors treated by PTT.

Water-soluble heparin–folic acid nanoparticles (*Fig. 6B*) were shown to bind the water-insoluble dye IR780 [58]. Water-insoluble folic acid molecules form a hydrophobic core, with IR780 incorporated in the particle center, while heparin molecules form a hydrophilic layer on the particle surface. A small fraction of folic acid molecules are located on the particle surface, forming an address for targeting tumor cells expressing the folate receptor. These particles exhibit good monodispersity, high stability, and specificity for folate-positive MCF-7 cells. *In vivo *experiments have demonstrated that folic acid–heparin particles not only exert a photothermal effect upon irradiation, but also serve as a tool to visualize the tumor focus.

Another iodinated analog of ICG, DiR (1,1-dioctadecyl-3,3,3,3-tetramethylindotricarbocyanine iodide) absorbing at 808 nm, was used for IR visualization and simultaneous photothermal ablation of breast cancer tumors and metastases [64]. The dye is passively delivered in polymeric nanoparticles to inflammatory foci. DiR possesses both photothermal and photodynamic properties: injection of the dye directly into a tumor, followed by irradiation, causes the destruction of cancer cells through the simultaneous generation of heat and reactive oxygen species by the dye [111].

The cyanine dye CySCOOH, which is produced by introducing a rigid cyclohexenyl ring into the heptamethine chain of ICG (*Table* and *Fig. 5*), conjugated with HSA (*Fig. 6C*), showed improved accumulation and longer retention in a tumor compared to the free dye CySCOOH. *In vitro *and *in vivo *experiments demonstrated that the dye could be used for photoacoustic imaging, IR fluorescence bioimaging, and thermal therapy [91]. In *in vivo *experiments, complete photothermal tumor ablation was achieved with a single intravenous injection of the drug, followed by IR irradiation (808 nm, 1 W/cm2, 5 min).

The carbocyanine dye Cypate is another cyanine dye that absorbs in the near IR region (~800 nm, *Table*) and exhibits photoacoustic and photothermal effects upon irradiation [81, 112]. Protein-coated gadolinium nanoparticles were used to deliver this dye (*Fig. 6D*) [113]. The dye molecules were covalently attached to the protein shell using a carbodiimide reaction. *In vivo *experiments demonstrated that these nanoparticles perfectly visualize the tumor focus by photoacoustic, magnetic resonance, and fluorescent imaging, passively accumulate in tumor cells, and cause complete photothermal tumor ablation after one phototherapy session.

## CONCLUSION

Photothermal therapy of tumor neoplasms using near-infrared organic dyes is an actively developing and promising area of biomedicine. Thanks to the relatively low (compared to other photothermal agents) cost of the used dyes, their ability to passively accumulate in tumors, the possibility of housing them in a wide range of nanocarriers for active delivery (including targeted delivery), and thanks to the minimal invasiveness of the treatment and minor side effects in comparison with inorganic photothermal agents, organic dyes have been attracting increasing attention from researchers. To improve the biocompatibility and enhance the phototheranostic properties of indocyanine dyes, along with the development of new modifications of the dyes, new methods for their delivery by nanoagents are being developed.

The ability of photoactivated dyes for multimodal imaging, e.g., simultaneous infrared fluorescence and photoacoustic imaging, makes them choice agents for cancer phototheranostics. An area of growth in this research field is the development of multifunctional nanoplatforms that combine the ability, when irradiated, not only to fluoresce, but also to exhibit photothermal and/or photodynamic properties. The multimodal nanoplatforms described in this review enable not only therapy that combines different therapeutic approaches leading to impressive synergistic effects, but also simultaneous visualization of disease foci, as well as non-invasive monitoring of the response to treatment.

The particular attention of researchers is focused on the development of targeted drugs that can minimize the adverse toxicity and side effects of cancer therapy. At present, this direction is rapidly developing not only thanks to the use of traditional antibodies, but also thanks to new targeted non-immunoglobulin scaffolds (affibodies, anticalins, designed ankyrin repeat proteins, etc.).

In the opinion of these authors, the development of similar multimodal theranostic nanoplatforms will represent the leading edge of experimental oncology, enabling solutions to the most vexing problems of non-invasive diagnostics, highly effective precision treatment, and real-time monitoring of treatment efficacy.

## Abbreviations

BSA: bovine serum albumin
HSA: human serum albumin
ICG: indocyanine green
IR: infrared
PDT: photodynamic therapy
PEG: polyethylene glycol
PEI: polyethyleneimine
PLGA: polylactide glycolide
PTT: photothermal therapy
PTX: paclitaxel
RB: Rose Bengal
ROS: reactive oxygen species
UNP: upconverting nanoparticle
